# Factors influencing depressive symptoms in Chinese female breast cancer patients: a meta-analysis

**DOI:** 10.3389/fpsyg.2024.1332523

**Published:** 2024-04-10

**Authors:** Qingyuan Zhang, Gen Wu, Jianfei Chen, Kui Fang, Qianqian Liu, Pan Zhang, Hongzhen Zhu, Chunhua Zhang

**Affiliations:** ^1^Zhongnan Hospital of Wuhan University, Wuhan, Hubei, China; ^2^Center of Structural Cardiology, Zhongnan Hospital, Wuhan University, Wuhan, Hubei, China; ^3^The Fifth Affiliated Hospital of Southern Medical University, Guangzhou, Guangdong, China; ^4^Wuhan University, Wuhan, Hubei, China; ^5^The First Hospital of China Medical University, Shenyang, Liaoning, China

**Keywords:** breast cancer, depression, related factors, meta-analysis, female patients

## Abstract

**Objective:**

To systematically evaluate and explore the factors influencing depressive symptoms in female breast cancer patients in China through meta-analysis.

**Methods:**

Relevant data were retrieved from cross-sectional studies or cohort studies on depressive symptoms of Chinese breast cancer within the following databases: PubMed, Embase, Cohrane Library, Web of 105 Science, Database of Medical Literature (CBM), Wan Fang Data, CNKI, and VIP databases. The literature screening, data extraction and literature quality evaluation were performed by two researchers by carefully reading the title, abstract and full text, and meta-analysis was performed using Stata 1.5 software after extracting relevant data.

**Results:**

Fourteen papers were finally included, with a cumulative total of 3,071 people surveyed, and a total of 1,298 breast cancer patients were detected with depression, with a detection rate of depressive symptoms of 42.26%; meta analysis showed that age less than 40 years old, unmarried, less than undergraduate education, monthly income <5,000 yuan, advanced breast cancer, radical breast cancer surgery, family history, living in rural areas, underlying disease stage and chemotherapy were associated with an increased incidence of depression in breast cancer patients.

**Conclusion:**

The detection rate of depressive symptoms in female breast cancer patients is high, and there is a need to strengthen depression-related psychological screening of breast cancer patients and provide them with individualized interventions to reduce the incidence of depression in breast cancer patients and to lower the level of depression already present in the patients.

## 1 Introduction

According to the 2020 global cancer statistics reported by the International Agency for Research on Cancer (IARC), breast cancer has replaced lung cancer as the most common malignant tumor in the world, with 2.26 million new cases (Sung et al., 2021). While breast cancer survival rates have improved with modern medical technology, psychological problems abound (Jacob et al., 2016). Both the diagnosis of breast cancer itself and its treatment can lead to or exacerbate a patient’s mental health condition (Epping-Jordan et al., 1999; Drageset et al., 2012), depression is the most common form of psychological distress (Loeffler et al., 2018; Civilotti et al., 2020). According to Tsaras et al. the prevalence of depression in breast cancer patients was 38.2% (Tsaras et al., 2018). The results of a meta-analysis showed that the prevalence of depression in breast cancer patients was 32.2% (Pilevarzadeh et al., 2019). Many breast cancer patients experience high levels of psychological problems, such as depression, and this can have a negative impact on survival rates and quality of life for patients (Setyowibowo et al., 2022). In recent years, the depressive state of breast cancer patients has gradually attracted the attention of scholars at home and abroad, but the influencing factors of depression are still unclear and lack of relevant systematic evaluation in China. In this study, the results of published studies on factors related to depression in Chinese women with breast cancer were analyzed using meta-analysis to understand the influencing factors of depression in breast cancer and to lay the foundation for individualized interventions for breast cancer survivors with depressive symptoms.

## 2 Information and methods

### 2.1 Inclusion and exclusion criteria of the literature

Inclusion criteria: (1) The study subjects were Chinese female breast cancer patients with a clear diagnosis; (2) A scale was used to assess the degree of depressive symptoms in breast cancer patients; (3) The types of studies were cross-sectional, cohort, and case–control studies; (4) All retrievable literature in Chinese and English. Exclusion criteria: (1) literature with data that could not be extracted or transformed; (2) duplicate publications; (3) conference abstracts or reviews; and (4) low quality of literature: an AHRQ [Agency for Healthcare Research and Quality] score ≤ 3 (Zeng et al., 2012).

Search strategy using “breast cancer/breast cancer/breast tumor” and “depression/depressive symptoms” and “prevalence/detection rate/status” as the search terms, the Chinese search strategy was (“breast cancer” OR “breast cancer” OR “breast tumor”) AND ((“depression” OR “breast cancer” OR “breast tumor”) AND ((“depression” OR “breast cancer” OR “breast tumor”)). “Breast Cancer” OR “Breast Cancer” OR “Breast Tumor”)AND((“Depression” OR “depressive symptoms”) AND (“prevalence” detection rate “status”)), and the English search strategy was (“breast neoplasm*” OR “breast tumor*” OR “breast cancer “OR “Mammary cancer “) AND (“Depressive Symptom” OR “Depression”) AND (“Detection Rate “OR “Epidemiology” OR “Prevalence”). AND (“Chinese “OR “China”). Computerized search of English and Chinese databases: PubMed, Embase, Cohrane Library, Web of Science, Database of Medical Literature (CBM), Wan Fang Data, CNKI, and VIP databases. The search was conducted for studies on the occurrence of depressive symptoms in Chinese breast cancer patients before July 20, 2023, using a combination of breast cancer-related search terms and depression-related search terms.

### 2.2 Literature screening and data extraction

Literature screening and data extraction were performed by two researchers who independently screened the literature according to the inclusion and exclusion criteria using Endnote software, cross-checked the extracted data, and in the event of disagreement, a third researcher adjudicated. Information such as author, publication date, region, age, assessment tool, sample size, depressive symptom detection rate, cancer stage and influencing factors were extracted from the literature.

### 2.3 Quality evaluation of the included literature

Two researchers evaluated the risk of bias of the included studies by using the AHRQ quality evaluation scale, which consists of 11 items, with a score of 1 for a “yes” answer and 0 for a “no” or “unclear” answer. The scale consists of 11 items, with 1 point for “yes” and 0 points for “no” or “unclear.” The range goes from 0 to 11, where 0–3 is low quality, 4–7 is medium quality, and 8–11 is high quality (Zeng et al., 2012).

### 2.4 Statistical methods

Meta-analysis was performed using Stata 1.5 software. Effect sizes were described using ORs of factors related to depressive symptoms in breast cancer patients and their 95% CIs. The heterogeneity test was used to determine whether there was heterogeneity among the studies in the included literature; if *p* > 0.1 and *I*^2^ < 50%, suggesting that there was no obvious statistical heterogeneity among similar studies, a fixed-effects model was chosen; if *p* < 0.1 and *I*^2^ > 50%, suggesting that there was statistical heterogeneity, a random-effects model was chosen for the merger analysis; and by comparing the difference between the fixed-effects model and the merger of random-effects model, a sensitivity analysis was performed.

## 3 Results

### 3.1 Literature search results

**Figure 1:**
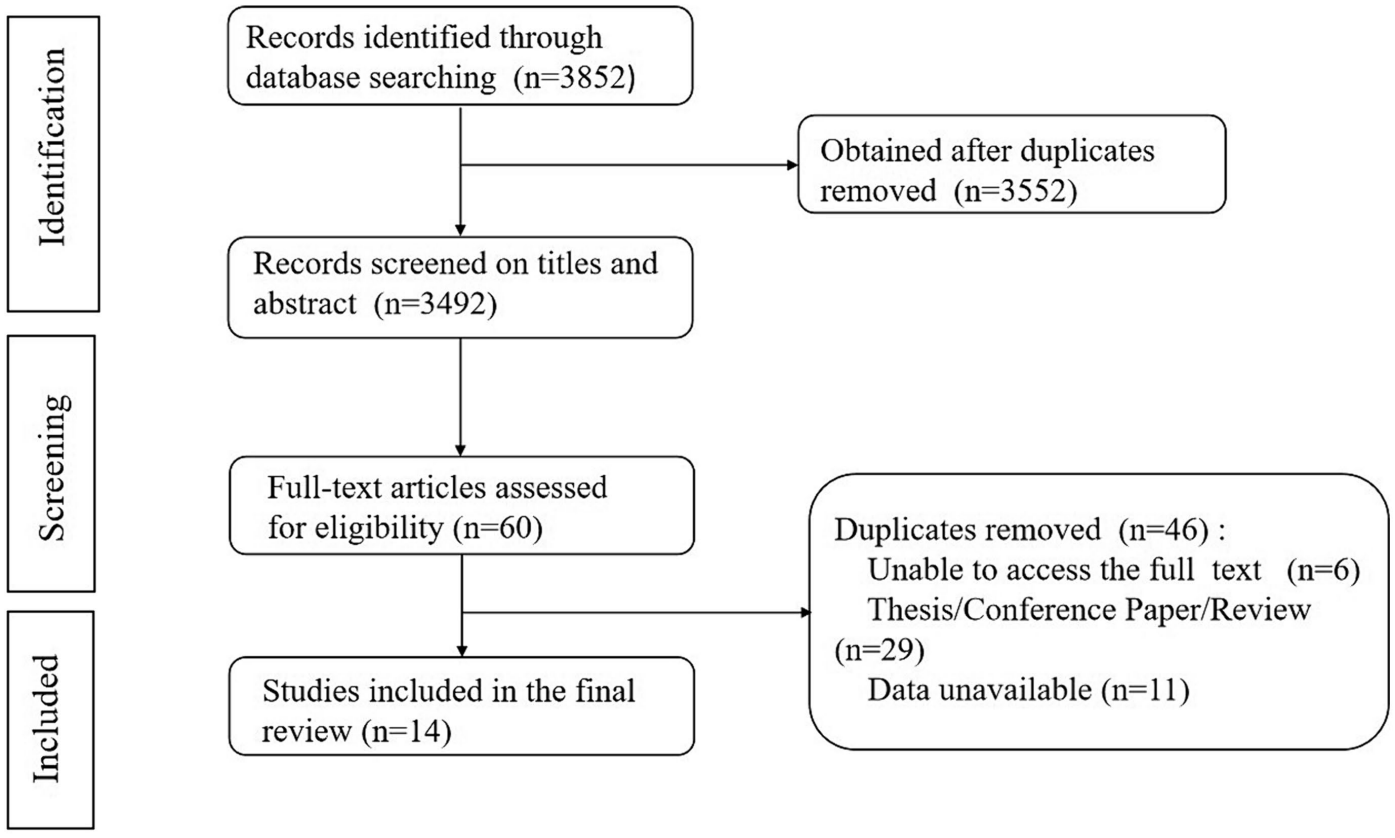
The flow chat of the identification of relevent studies.

After the initial search of 3,852 articles, Endnote X9 software was applied to exclude 300 duplicates, and 3,552 articles were obtained. After reading the topics and abstracts of the literature, 3,492 articles were obtained, and after reading the full text of the literature, 14 articles were finally included in the study after evaluation of the quality of the literature, with a total of 3,071 patients. The entire process is shown in Figure 1.

### 3.2 Basic characteristics of the included literature

Fourteen literatures published in 2014–2021 were finally included, with a cumulative total of 3,071 patients; five studies were conducted in the north and nine in the south, covering a total of nine provinces across the country, with a total of 1,298 breast cancer patients detected with depression, and an overall detection rate of depressive symptoms of 42.26%. The basic characteristics are shown in Table 1.

**Table 1:** Basic characteristics of included studies.

Author (year)	District	Assessment tools	Sample size	Disease rate	Age	Cancer staging	Factor	Quality assessment
Liu et al. (2014)	Guangzhou, Guangdong	SDS	245	120	57.2 ± 11.7	I ~ III	3, 7, 9	8
Wang and Xue (2014)	Hebei Chengde	HAMD	486	184	53.4 ± 12.5	0 ~ I II ~ III	2, 3, 4	5
Xu et al. (2015)	Guangdong Shenzhen	BDI	205	48	46.4 ± 8.6	–	2, 3, 4, 7, 8, 9, 11, 12	6
Liu (2016)	Ji’nan, Shandong	SDS	151	89	≥18	I ~ IV	2, 7, 9, 10	6
Lv et al. (2017)	Ji’nan, Shandong	HAMD; CES-D	180	112	43.0 ± 8.2	–	2, 3, 4, 6, 8	6
Yang (2017)	Chengdu, Sichuan	SDS	123	36	57.1 ± 10.2	I ~ II	8,	6
Zhu et al. (2017)	Changsha, Hunan	SDS	373	142	≥20	I ~ IV	7	6
Bai et al. (2018)	Beijing	SDS	137	65	47.2 ± 10.6	–	1, 2, 4, 10	7
Jin et al. (2018)	Nanjing, Jiangsu	HAMD	233	82	20 ~ 45	–	1, 2, 3, 5, 8, 9, 12	6
Xu et al. (2019)	Zhengzhou, Henan	SDS	130	80	46.7 ± 5.6	I ~ II III ~ IV	2, 4, 5, 6, 7, 9	8
Chen et al. (2019)	Jingzhou, Hubei	HAMD; CES-D	210	102	≥18	–	1, 2, 3, 5, 6, 11	7
Wang et al. (2020)	Henan Zhumadian	SDS	140	89	≥18	I II	1, 2, 4, 6	6
Lv et al. (2020)	Langfang, Hebei	SDS	222	88	58.6 ± 4.6	I ~ II III ~ IV	1, 2, 4, 6, 7	6
Chen et al. (2021)	Zhaoqing of Guangdong	SDS	236	61	41.8 ± 3.5	I ~ IV	2, 4, 5, 6, 7, 9, 10	6

Influencing factors: 1, age; 2, marriage; 3, occupation; 4, education; 5, economy; 6, mode of payment; 7, stage of disease; 8, mode of surgery; 9, family history; 10, place of residence; 11, underlying disease; 12, chemotherapy. Measurement tools: SDS, Self-Depression Scale; BDI, Beck Depression Self-Assessment Questionnaire; HAMD, Hamilton Depression Scale; HADS, Hospital Anxiety Depression Scale; PHQ-9, Patient Health Questionnaire Depression Scale; CES-D, Center for Epidemiologic Studies Depression Scale. “–” indicates that this item is not available.

### 3.3 Literature quality evaluation

The 14 included literature were of medium to high quality, all articles were cross-sectional studies and were evaluated according to the AHRQ recommendations; two (Liu et al., 2014; Xu et al., 2019) literature were evaluated as 8 “Yes”; two (Bai et al., 2018; Chen et al., 2019) literature were evaluated as 7 “Yes “; one (Wang and Xue, 2014) literature evaluated with 5 “yes”; and the remaining 9 literature (Xu et al., 2015; Liu, 2016; Lv et al., 2017, 2020; Yang, 2017; Zhu et al., 2017; Jin et al., 2018; Wang et al., 2020; Chen et al., 2021) evaluated with 6 “yes” Table 2.

**Table 2:** Risk of bias ratings for cross-sectional studies.

First author	Q1	Q2	Q3	Q4	Q5	Q6	Q7	Q8	Q9	Q10	Q11	Totals
Liu et al. (2014)	1	1	1	0	1	1	1	1	1	0	0	8
Wang and Xue (2014)	1	1	1	0	0	1	0	1	0	0	0	5
Xu et al. (2015)	1	1	1	0	0	1	0	1	1	0	0	6
Liu (2016)	1	1	1	0	0	0	0	1	1	1	0	6
Lv et al. (2017)	1	1	1	0	0	1	0	1	0	1	0	6
Yang (2017)	1	1	1	0	0	1	0	0	1	1	0	6
Zhu et al. (2017)	1	1	1	0	0	1	0	0	1	1	0	6
Bai et al. (2018)	1	1	1	0	0	1	1	0	1	1	0	7
Jin et al. (2018)	1	1	1	0	0	0	1	0	0	1	1	6
Xu et al. (2019)	1	1	1	0	0	1	1	1	1	1	0	8
Chen et al. (2019)	1	1	1	0	0	1	0	1	1	1	0	7
Wang et al. (2020)	1	1	1	0	0	1	0	1	0	1	0	6
Lv et al. (2020)	1	1	1	0	0	1	0	1	1	0	0	6
Chen et al. (2021)	1	1	1	1	0	1	0	1	0	0	0	6

1. Is the source of information (survey, literature review) clearly identified? 2. Are inclusion and exclusion criteria for exposed and non-exposed groups (cases and controls) listed or referenced in previous publications? 3. Is the time period for identifying patients given? 4. Are the study subjects consecutive, if not a population source? 5. Are the evaluator’s subjective factors overshadowing any other aspects of the subject matter of the study? 6. Describes any assessments for quality assurance (e.g., testing/retesting of primary outcome indicators). 7. Explains the rationale for excluding any patients from the analysis. 8. Describes how measures of confounding were evaluated and/or controlled. 9. If possible, explains how missing data were handled in the analysis? 10. Summarizes the response rate of the patients and the completeness of the data collection. 11. If there was follow-up, identify the expected percentage of patients with incomplete data or the outcome of follow-up.

### 3.4 Meta-analysis results

The results of the heterogeneity test showed that there was significant heterogeneity among the literature related to each factor of age, marriage, education, disease stage, place of residence, chemotherapy, and the presence of family history (*p* < 0.1, *I*^2^ > 50%), so the random effects model was used to merge the effect sizes; the heterogeneity of the results of combining the factors of influence such as occupation, economy, mode of payment, surgical method, and the presence of underlying diseases was less heterogeneous (*p* > 0.1, *I*^2^ < 50%), so the fixed-effects model was used to combine effect sizes. Meta analysis showed that age less than 40 years old, unmarried, less than undergraduate education, monthly income less than 5,000 yuan, advanced breast cancer, radical breast cancer surgery, family history, living in rural areas, underlying disease stage and chemotherapy were associated with an increased incidence of depression in breast cancer patients (Table 3).

**Table 3:** Meta-analysis results of influencing factors of depressive symptoms in breast cancer patients.

Influencing factor		Control group	Literature count	I2	*Q* valve	p	Meta-analysis model	OR (95%CI)
Age	<40	≥40	5	59.3%	7.37	0.061	Random-effects	1.45 (0.87 ~ 2.43)
Matrimony	Getting married	Not married	11	60.0%	25.03	0.005	Random-effects	0.62 (0.41 ~ 0.94)
Occupation	Employed	Not employed	6	21.6%	5.10	0.277	Common-effects	0.59 (0.46 ~ 0.76)
Educational level	College degree or above	Below associate degree	8	51.3%	14.38	0.045	Random-effects	0.88 (0.64 ~ 1.21)
Economy	≤5 k	>5 k	4	34.3%	3.04	0.218	Common-effects	1.58 (1.08 ~ 2.30)
Payment method	reimbursable	Self-funded	6	6.0%	5.32	0.378	Common-effects	0.17 (0.10 ~ 0.29)
Stage of disease	0 ~ 2	3 ~ 4	7	57.6%	14.14	0.028	Random-effects	0.54 (0.36 ~ 0.81)
Mode of operation	Radical operation	Breast-conserving surgery	5	37.3%	6.38	0.172	Common-effects	1.36 (1.00 ~ 1.86)
Family history	Yes	Not	6	69.4%	13.06	0.011	Random-effects	1.11 (0.50 ~ 2.47)
Place of residence	city	Non-urban	4	61.0%	7.70	0.053	Random-effects	0.84 (0.48 ~ 1.49)
Underlying disease	Yes	Not	2	30.4%	1.44	0.231	Common-effects	1.08 (0.66 ~ 1.77)
chemotherapy	Yes	Not	2	0.0%	0.54	0.463	Common-effects	1.43 (0.84 ~ 2.45)

### 3.5 Sensitivity analysis

Sensitivity analyses of the 12 factors included were conducted using the fixed-effects model and random effects respectively, and the results showed that the combined ORs of the two models and their 95% CIs were relatively close, indicating that the meta-analysis of this study was stable (Table 4).

**Table 4:** Sensitivity analysis of factors influencing depressive symptoms in breast cancer patients.

Research factors	Common-effects OR (95%CI)	Random-effects OR (95%CI)
Age	1.47 (1.06 ~ 2.03)	1.45 (0.87 ~ 2.43)
Matrimony	0.61 (0.47 ~ 0.78)	0.62 (0.41 ~ 0.94)
Occupation	0.59 (0.46 ~ 0.76)	0.58 (0.44 ~ 0.78)
Educational level	0.88 (0.71 ~ 1.09)	0.88 (0.64 ~ 1.21)
Economy	1.58 (1.08 ~ 2.30)	1.56 (0.97 ~ 2.53)
Payment method	0.17 (0.10 ~ 0.29)	0.17 (0.09 ~ 0.30)
Stage of disease	0.58 (0.46 ~ 0.75)	0.54 (0.36 ~ 0.81)
Mode of operation	1.36 (1.00 ~ 1.86)	1.34 (0.89 ~ 2.00)
Family history	0.89 (0.58 ~ 1.35)	1.11 (0.50 ~ 2.47)
Place of Residence	0.84 (0.59 ~ 1.19)	0.84 (0.48 ~ 1.49)
Underlying disease	1.08 (0.66 ~ 1.77)	1.09 (0.61 ~ 1.96)
chemotherapy	1.43 (0.84 ~ 2.45)	1.45 (0.84 ~ 2.50)

## 4 Discussion

Breast cancer is the most common malignant tumor in women, and the incidence rate is not only high but also on the rise year by year, which seriously affects the physical and mental health of women (Sung et al., 2021). The diagnosis of breast cancer itself not only leads to severe stressful psychological events, but also patients have to experience a series of adverse reactions such as pain, treatment side effects, and body image changes in the course of disease treatment, so most of the patients will experience different degrees of psychological disorders (Fortin et al., 2021). A review showed that >12% of women develop depressive symptoms after diagnosis of breast cancer (Fann et al., 2008). Depression not only affects patients’ psychological functioning, physiological functioning, treatment adherence, and quality of life, but is also an important factor contributing to the occurrence of mortality in breast cancer patients (Watson et al., 2005; Shim et al., 2020). Meta-analysis has shown that there is a significant correlation between depression and mortality in breast cancer (Satin et al., 2009; Giese-Davis et al., 2011), and meta-analysis by He et al. has shown that the prevalence of depression in female breast cancer patients in China is as high as 42.1% (He et al., 2023), seriously affecting the prognosis and quality of survival of patients, in order to prevent and control this mental health problem, clinical practitioners should identify the prevalent factors in patients at an early stage so that timely and effective interventions can be taken.

The discovery of risk factors for depression in breast cancer patients is an important condition for improving the psychological status of patients, and in this study, we found that age < 40 years, poor economic status, surgical methods, and the presence of genetic and family history are risk factors for depression in female breast cancer patients, which is in line with the findings of Puigpinós-Riera et al. (2018), the reason being that young and unmarried women are highly concerned about their self-image, focusing on their own appearance, and undergoing mastectomy is a great blow to them, with a decrease in self-esteem and self-efficacy, and fear that the disease will affect their marriages and childbearing, family stability, and careers (Shen et al., 2018). Patients with a family history and underlying disease tend to worry about prognosis and fear (Bayer et al., 2022).

The present study found that marital and employment status was a protective factor for the occurrence of depression in breast cancer patients, married and employed breast cancer patients are significantly less likely to develop depression, consistent with the findings of Casavilca-Zambrano et al. (2020) that employed and married patients were less likely to experience depressive symptoms, which was associated with higher emotional, family and economic levels of the patients. A previous study of 1,400 Chinese women found that employed and high-income women were less likely to experience depressive symptoms and they had a higher sense of social support (Chen et al., 2009). Patients with higher education also had a fuller understanding of the disease and better individual physical and mental regulation, and were less likely to experience negative emotions. Patients whose payments were covered by health insurance or commercial insurance had relatively less financial stress and consequently lower psychological stress (Li et al., 2019). The lower the tumor stage, the lower the likelihood of malignancy, metastasis, and recurrence, the higher the survival rate, and the significantly lower the occurrence of somatic symptoms and negative emotions in patients (Niu et al., 2019). Patients living in rural areas may be related to their “country-style” lifestyle, in which they try to see the positive outcome of the disease when facing breast cancer and are grateful for the possibility of treatment, maintaining optimism and reducing the worry of family members, and experiencing relatively little depression (Schlegel et al., 2009). Adverse effects such as nausea and vomiting occur in most patients during intravenous chemotherapy and chemotherapy as a risk factor in this study is consistent with the findings of Hajj et al. (2021) and others, but in Henselmans et al. (2010) and others reported a large group of women who did not experience mood disorders during chemotherapy. Therefore, further studies are needed to determine this variable.

The limitations of this study include: (1) the number of included literatures is not large enough and the sample size is small; the research design and analysis methods of the included studies vary greatly, which has some influence on the results; (2) the number of literatures included for individual influencing factors is small, which may have some influence on the conclusions of the meta-analysis; (3) a total of 14 literatures included in the present study involve six depression scales, and the survey entries for the different depression scales are different, and there may be a difference in the detection rate of the different scales, which may affect the results of the study; and (4) the present study is a cross sectional study conducted by means of the form of scales, and the individuals have not been subjected to a comprehensive psychological interview, which is not sufficiently comprehensive to investigate and analyze the influencing factors.

## 5 Conclusion

In summary, advanced age, poor economic status, radical mastectomy, family history, ongoing disease, and chemotherapy may increase the incidence of depression in breast cancer patients. Patients should undergo early prevention and timely intervention for the above risk factors after diagnosis of breast cancer, which plays an important role in decreasing the incidence and severity of depression.

## Data availability statement

The original contributions presented in the study are included in the article/supplementary material, further inquiries can be directed to the corresponding author/s.

## Author contributions

QZ: Writing – original draft. GW: Writing – review & editing. JC: Writing – original draft. KF: Writing – original draft. QL: Writing – original draft. PZ: Writing – original draft. HZ: Writing – original draft. CZ: Writing – review & editing, Funding acquisition.

## References

[ref1] Bai L. Yi S. Bai W. Liu W. Lu Y. Zhang T. . (2018). Investigation of the detection rate of depression and its risk factors in 137 breast cancer patients. Mil. Med. 42, 398–399. doi: 10.7644/j.issn.1674-9960.2018.05.018

[ref2] Bayer S. J. Yang G. S. Lyon D. E. (2022). Genetic variation associated with depressive symptoms in breast Cancer patients: a systematic review. Cancer Nurs. 45, E197–e205. doi: 10.1097/NCC.0000000000000903, PMID: 33156013

[ref3] Casavilca-Zambrano S. Custodio N. Liendo-Picoaga R. Cancino-Maldonado K. Esenarro L. Montesinos R. . (2020). Depression in women with a diagnosis of breast cancer. Prevalence of symptoms of depression in Peruvian women with early breast cancer and related sociodemographic factors. Semin. Oncol. 47, 293–301. doi: 10.1053/j.seminoncol.2020.08.003, PMID: 33046263

[ref4] Chen Y. Li G. He Y. (2021). Study on psychological status and influencing factors of breast cancer patients in western Guangdong. South China Prevent. Med. 47, 540–542. doi: 10.12183/j.scjpm.2021.0540

[ref5] Chen J. Wang H. Ding J. Zhang H. (2019). Relationship between subthreshold depressive status and quality of social relationships in patients with first diagnosis of breast cancer. China Med. Herald 16, 37–40. doi: CNKI:SUN:YYCY.0.2019-02-010

[ref6] Chen X. Zheng Y. Zheng W. Gu K. Chen Z. Lu W. . (2009). Prevalence of depression and its related factors among Chinese women with breast cancer. Acta Oncol. 48, 1128–1136. doi: 10.3109/02841860903188650, PMID: 19863220 PMC3771388

[ref7] Civilotti C. Acquadro Maran D. Santagata F. Varetto A. Stanizzo M. R. (2020). The use of the distress thermometer and the hospital anxiety and depression scale for screening of anxiety and depression in Italian women newly diagnosed with breast cancer. Support Care Cancer 28, 4997–5004. doi: 10.1007/s00520-020-05343-x, PMID: 32036468

[ref8] Drageset S. Lindstrøm T. C. Giske T. Underlid K. (2012). "The support I need": women's experiences of social support after having received breast cancer diagnosis and awaiting surgery. Cancer Nurs. 35, E39–E47. doi: 10.1097/NCC.0b013e31823634aa 22134160

[ref9] Epping-Jordan J. E. Compas B. E. Osowiecki D. M. Oppedisano G. Gerhardt C. Primo K. . (1999). Psychological adjustment in breast cancer: processes of emotional distress. Health Psychol. 18, 315–326. doi: 10.1037/0278-6133.18.4.315 10431932

[ref10] Fann J. R. Thomas-Rich A. M. Katon W. J. Cowley D. Pepping M. McGregor B. A. . (2008). Major depression after breast cancer: a review of epidemiology and treatment. Gen. Hosp. Psychiatry 30, 112–126. doi: 10.1016/j.genhosppsych.2007.10.008, PMID: 18291293

[ref11] Fortin J. Leblanc M. Elgbeili G. Cordova M. J. Marin M. F. Brunet A. (2021). The mental health impacts of receiving a breast cancer diagnosis: a meta-analysis. Br. J. Cancer 125, 1582–1592. doi: 10.1038/s41416-021-01542-3, PMID: 34482373 PMC8608836

[ref12] Giese-Davis J. Collie K. Rancourt K. M. Neri E. Kraemer H. C. Spiegel D. (2011). Decrease in depression symptoms is associated with longer survival in patients with metastatic breast cancer: a secondary analysis. J. Clin. Oncol. 29, 413–420. doi: 10.1200/JCO.2010.28.4455, PMID: 21149651 PMC3058287

[ref13] Hajj A. Hachem R. Khoury R. Hallit S. ElJEBBAWI B. Nasr F. et al. (2021). Clinical and genetic factors associated with anxiety and depression in breast cancer patients: a cross-sectional study. BMC Cancer 21:872. doi: 10.1186/s12885-021-08615-9, PMID: 34330229 PMC8323303

[ref14] He J. Gao J. Bai D. Zhang H. Chen H. Gong X. (2023). Meta-analysis of the detection rate of depressive symptoms in Chinese female breast cancer patients. Chin J. Ment. Health 37, 116–121. doi: 10.3969/j.issn.1000-6729.2023.02.004

[ref15] Henselmans I. Helgeson V. S. Seltman H. de Vries J. Sanderman R. Ranchor A. V. (2010). Identification and prediction of distress trajectories in the first year after a breast cancer diagnosis. Health Psychol. 29, 160–168. doi: 10.1037/a0017806, PMID: 20230089

[ref16] Jacob L. Bleicher L. Kostev K. Kalder M. (2016). Prevalence of depression, anxiety and their risk factors in German women with breast cancer in general and gynecological practices. J. Cancer Res. Clin. Oncol. 142, 447–452. doi: 10.1007/s00432-015-2048-5, PMID: 26377737 PMC11819146

[ref17] Jin C. Wang B. Hu J. (2018). Analysis of risk factors for postoperative depression in young and middle-aged female breast cancer patients. Chin. J. Front. Med. 10, 139–142. doi: 10.12037/YXQY.2018.10-32

[ref18] Li T. Tan H. Chen Y. Jiang J. Xiong M. (2019). The relationship between psychological resilience and quality of life during chemotherapy in postoperative breast cancer patients and the analysis of its influencing factors. Cancer Prog. 17, 2343–2347. doi: 10.11877/j.issn.1672-1535.2019.17.19.30

[ref19] Liu W. (2016). A survey of depression in 151 breast cancer patients. Chin. Med. Herald 22, 36–37,49. doi: CNKI:SUN:HNZB.0.2016-16-015

[ref20] Liu Y. Z. Zhou Q. Xie Y. T. Chen S. M. Zeng Y. J. Luo X. Z. et al. (2014). A study on the anxiety and depression status of female breast cancer patients and its influencing factors in the community of Guangzhou City, China. South China Prevent. Med. 40, 7–11. doi: CNKI:SUN:GDWF.0.2014-01-003

[ref21] Loeffler S. Poehlmann K. Hornemann B. (2018). Finding meaning in suffering?-meaning making and psychological adjustment over the course of a breast cancer disease. Eur. J. Cancer Care (Engl) 27:e12841. doi: 10.1111/ecc.12841, PMID: 29575157

[ref22] Lu J. Xu G. Chen H. Gao D. Feng Z. (2020). Analysis of the occurrence of depression in breast cancer chemotherapy patients and its influencing factors. South China Prevent. Med. 46, 706–708. doi: 10.12183/j.scjpm.2020.0706

[ref23] Lu L. Zhang X. Wang X. Zhang J. (2017). A study of subthreshold depression and social participation in young and middle-aged postoperative breast cancer patients. J. Nurs. 32, 84–87. doi: 10.3870/j.issn.1001-4152.2017.06.084

[ref24] Niu L. Liang Y. Niu M. (2019). Factors influencing fear of cancer recurrence in patients with breast cancer: evidence from a survey in Yancheng, China. J. Obstet. Gynaecol. Res. 45, 1319–1327. doi: 10.1111/jog.13978, PMID: 31016820

[ref25] Pilevarzadeh M. Amirshahi M. Afsargharehbagh R. Rafiemanesh H. Hashemi S. M. Balouchi A. (2019). Global prevalence of depression among breast cancer patients: a systematic review and meta-analysis. Breast Cancer Res. Treat. 176, 519–533. doi: 10.1007/s10549-019-05271-3, PMID: 31087199

[ref26] Puigpinós-Riera R. Graells-Sans A. Serral G. Continente X. Bargalló X. Domènech M. . (2018). Anxiety and depression in women with breast cancer: social and clinical determinants and influence of the social network and social support (DAMA cohort). Cancer Epidemiol. 55, 123–129. doi: 10.1016/j.canep.2018.06.002, PMID: 29940418

[ref27] Satin J. R. Linden W. Phillips M. J. (2009). Depression as a predictor of disease progression and mortality in cancer patients: a meta-analysis. Cancer 115, 5349–5361. doi: 10.1002/cncr.24561, PMID: 19753617

[ref28] Schlegel R. J. Talley A. E. Molix L. A. Bettencourt B. A. (2009). Rural breast cancer patients, coping and depressive symptoms: a prospective comparison study. Psychol. Health 24, 933–948. doi: 10.1080/08870440802254613, PMID: 20205037

[ref29] Setyowibowo H. Yudiana W. Hunfeld J. A. M. Iskandarsyah A. Passchier J. Arzomand H. . (2022). Psychoeducation for breast cancer: a systematic review and meta-analysis. Breast 62, 36–51. doi: 10.1016/j.breast.2022.01.005, PMID: 35121502 PMC8819101

[ref30] Shen Y. Zhang J. Bu Q. . (2018). A longitudinal study of the level of psychological distress in breast cancer patients and the factors influencing it. China Nurs Manage 18, 617–622. doi: 10.3969/j.issn.1672-1756.2018.05.009

[ref31] Shim E. J. Lee J. W. Cho J. Jung H. K. Kim N. H. Lee J. E. . (2020). Association of depression and anxiety disorder with the risk of mortality in breast cancer: a National Health Insurance Service study in Korea. Breast Cancer Res. Treat. 179, 491–498. doi: 10.1007/s10549-019-05479-3, PMID: 31673880

[ref32] Sung H. Ferlay J. Siegel R. L. Laversanne M. Soerjomataram I. Jemal A. . (2021). Global Cancer statistics 2020: GLOBOCAN estimates of incidence and mortality worldwide for 36 cancers in 185 countries. CA Cancer J. Clin. 71, 209–249. doi: 10.3322/caac.21660, PMID: 33538338

[ref33] Tsaras K. Papathanasiou I. V. Mitsi D. Veneti A. Kelesi M. Zyga S. . (2018). Assessment of depression and anxiety in breast cancer patients: prevalence and associated factors. Asian Pac. J. Cancer Prev. 19, 1661–1669. doi: 10.22034/APJCP.2018.19.6.1661 PMID: 29938451 PMC6103579

[ref34] Wang L. Liu D. Chen K. (2020). Analysis of anxiety and depression status and related factors in breast cancer surgery patients. Int. J. Psychiatry 47, 1222–1225.

[ref35] Wang L. Xue F. (2014). Analysis of the occurrence of postoperative depression and related risk factors in women with breast cancer(Chinese article). Med. Clin. Res. 3, 529–531. doi: 10.3969/j.issn.1671-7171.2014.03.041

[ref36] Watson M. Homewood J. Haviland J. Bliss J. M. (2005). Influence of psychological response on breast cancer survival: 10-year follow-up of a population-based cohort. Eur. J. Cancer 41, 1710–1714. doi: 10.1016/j.ejca.2005.01.012, PMID: 16098457

[ref37] Xu Y. Zhang S. Zhang H. (2019). Postoperative depressive states and factors affecting them in breast cancer patients. Chin. J. Health Psychol. 27, 369–372. doi: CNKI:SUN:JKXL.0.2019-03-014

[ref38] Xu N. Zhang P. Zhou D. Liu C. Zhang Y. Wang Y. (2015). Survey on postoperative depression status of breast cancer patients and analysis of risk factors. Chin. J. Mod. Med. 25, 68–72. doi: 10.3969/j.issn.1005-8982.2015.34.016

[ref39] Yang X. (2017). Analysis of factors influencing depression psychology in breast cancer patients. Hebei Med. 23, 1423–1426.

[ref40] Zeng X. Liu H. Chen X. Leng W. (2012). Meta-analysis series IV: a quality assessment tool for observational studies. Chin. J. Evid. Based Cardiovasc. Med. 4, 297–299. doi: 10.3969/j.1674-4055.2012.04.004

[ref41] Zhu Q. Meng P. Yang L. Liu N. Tian C. Wang Y. (2017). Investigation and analysis of depression status and high-risk influencing factors of breast cancer patients. Oncol. Pharmacol. 7, 114–118. doi: 10.3969/j.issn.2095-1264.2017.01.23

